# C20orf27 Promotes Cell Growth and Proliferation of Colorectal Cancer via the TGFβR-TAK1-NFĸB Pathway

**DOI:** 10.3390/cancers12020336

**Published:** 2020-02-02

**Authors:** Jing Gao, Yang Wang, Weixia Zhang, Jing Zhang, Shaohua Lu, Kun Meng, Xingfeng Yin, Zhenghua Sun, Qing-Yu He

**Affiliations:** MOE Key Laboratory of Tumor Molecular Biology and Key Laboratory of Functional Protein Research of Guangdong Higher Education Institutes, Institute of Life and Health Engineering, Jinan University, Guangzhou 510632, China; GJ_3304502656@163.com (J.G.); wangyang8857@gmail.com (Y.W.); tobedonezwx@163.com (W.Z.); zj6410@jnu.edu.cn (J.Z.); Lu3020111@163.com (S.L.); mengk_123@126.com (K.M.); yinxf119@163.com (X.Y.); szh2009069@163.com (Z.S.)

**Keywords:** CRC, *C20orf27*, growth, proliferation

## Abstract

*Background*: Colorectal cancer (CRC) is a high incidence of malignant tumors that lacks highly effective and targeted drugs and thus it is in urgent need of finding new specific molecular targets. *Methods and Results*: In this study, by using WST-1 (Highly water-soluble tetrazolium salt-1) and colony formation assays, we found that *C20orf27* (chromosome 20 open reading frame 27), a functionally unknown protein, enhanced the growth and proliferation of CRC cells. The nude mouse tumor formation experiments verified that *C20orf27* promoted the growth of CRC. Signal pathway analysis identified the TGFβR-TAK1-NFĸB cascade as a mediator in *C20orf27*-induced CRC progression. Inhibition experiments using NFĸB inhibitors reversed this progression. Co-immunoprecipitation showed that *C20orf27* promoted the activation of the TGFβR-TAK1-NFĸB pathway by interacting with PP1c (the catalytic subunit of type 1 phosphatase). *Conclusions*: Our results firstly characterized the functional role and molecular mechanism of *C20orf27* in driving CRC growth and proliferation through the TGFβR-TAK1-NFĸB pathway, suggesting its potential as a novel CRC candidate therapeutic target and tumor marker.

## 1. Introduction

According to the latest statistics, colorectal cancer (CRC) has become the fourth most deadly cancer in the world, with nearly 900,000 deaths each year [1]. CRC accounts for approximately 10% of the total number of malignant tumors and tumor-related deaths worldwide each year [2]. It is expected that, by 2035, the new incidence of CRC in the world will reach 25 million [2,3]. Although many new therapies have doubled the overall survival of advanced CRC for three years [1], survival is still not ideal. Therefore, to increase the rate of early detection and to reduce the incidence and mortality of CRC, it is necessary to further identify key genes as tumor markers and molecular targets for the diagnosis and therapy of CRC.

The complete sequencing program for the entire human genome, the Human Genome Project (HGP), was launched in 1990, and then was completed in 2003 [4]. Although this is an advancement for the deep understanding of the human genome, there are still many more in-depth questions that have not been answered. Therefore, a new project was proposed later to understand the functional roles of the genes and their encoded proteins. The Encyclopedia of DNA Elements (ENCODE) project was launched in September 2003, with the goal of explaining the sequencing results obtained by HGP [4]. Through the use of bioinformatics methods and experiments, it is possible to analyze gene function and mode of action, laying the foundation for further explanation of the etiology of genetic diseases and for molecule-targeted therapy of malignant tumors. 

*C20orf27* is a functionally unknown gene on chromosome 20 in the human genome. Three tissue microarray results revealed that its expression in CRC cells is higher than that in normal intestinal epithelial cells [5,6,7]. However, the phenotypic and functional mechanisms of *C20orf27* have never been studied. In this work, we aimed to investigate the role of *C20orf27* in CRC and to explore its regulatory mechanisms. Our results indicate that *C20orf27* promotes the growth and proliferation of CRC cells by increasing the expression of NFĸB. By interacting with PP1c (the catalytic subunit of type 1 phosphatase), *C20orf27* activates the TGFβR-TAK1-NFĸB pathway. Our research provides new ideas and targets for precision medicine for the treatment of CRC.

## 2. Results

### 2.1. C20orf27 Promotes the Growth and Proliferation of CRC Cells

We focused on 24 functionally unknown genes on chromosome 20 of the human genome. By searching on the Oncomine website, we found that 8 genes among the 24 genes were highly expressed in CRC based on the previously reported tissue microarray results. We compared the transcription levels of these eight genes in eight CRC cells and one normal intestinal epithelial cell (NCM460) using real-time qRT-PCR (Figure S1A). We selected PRNP (prion protein) and *C20orf27* with higher transcription levels in cancer cells than normal intestinal epithelial cells, and constructed overexpressing stable cells to evaluate their ability in promoting cell proliferation using WST-1 assay (Figure S1B–E). Then, we selected *C20orf27*, which significantly promoted the proliferation of CRC cells, as the target gene to investigate its in-depth functional mechanism.

**Figure 1:**
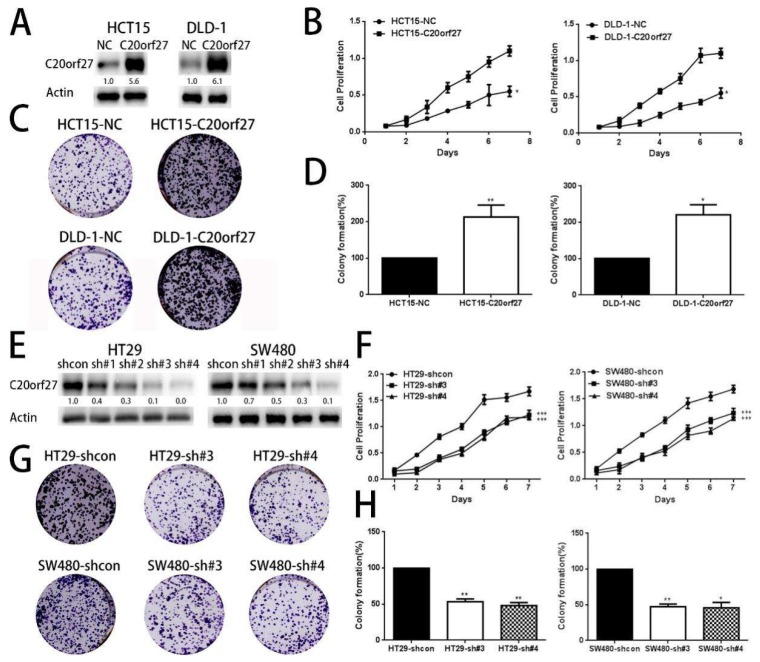
*C20orf27* promotes the growth and proliferation of CRC cells in vitro. (**A**) Overexpression of *C20orf27* in HCT15 and DLD-1 cell lines was confirmed using Western blot analysis; (**B**) Changes in absorbance after overexpression of *C20orf27* in HCT15 and DLD-1 cells as detected by WST-1 (Highly water-soluble tetrazolium salt-1) assay; (**C**) and (**D**) Clone formation assays in overexpressing *C20orf27* and control cells of HCT15 and DLD-1; (**E**) *C20orf27* silencing in SW480 and HT29 cell lines was confirmed by Western blot analysis; (**F**) Changes in absorbance after *C20orf27* silencing in SW480 and HT29 cells as detected by WST-1 assay; (**G**) and (**H**) Clone formation assays in *C20orf27* silencing and their control cells of SW480 and HT29. * *p* < 0.05, ** *p* < 0.01, *** *p* < 0.001.

Western blot analysis was used to detect the protein expression levels of *C20orf27* in eight CRC cells and one normal intestinal epithelial cell (Figure S2A). The expression of *C20orf27* in colorectal cancer tissues is obviously higher than that of adjacent tissues (Figure S2B). HCT15 and DLD-1 cells with low expression of *C20orf27* were used for the construction of stable overexpressing cell lines (Figure 1A). WST-1 analysis showed that *C20orf27* overexpression significantly increased cell mitochondrial dehydrogenase activity as compared to controls (Figure 1B). Colony formation experiments showed that *C20orf27* overexpression increased cell colony formation in HCT15 and DLD-1 cells (Figure 1C,D). In reverse, we used SW480 and HT29 cells with high levels of *C20orf27* for silencing experiments. The results showed that mitochondrial dehydrogenase activity (Figure 1F) and cloning ability were inhibited after *C20orf27* silencing (Figure 1E) as compared to controls (Figure 1G,H). These data indicate that *C20orf27* promotes the growth and proliferation of CRC cells.

### 2.2. C20orf27 Regulates Cell Cycle and Apoptosis via NFĸB Pathway

**Figure 2:**
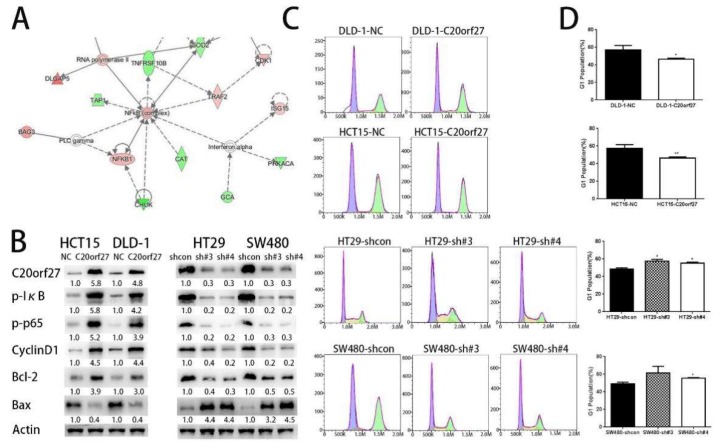
*C20orf27* regulates the cell cycle by activating the NFĸB pathway. (**A**) IPA analysis of differentially expressed proteins between *C20orf27*-overexpressing HCT15 /DLD-1 and HCT15 /DLD-1-NC cells, suggesting the NFĸB pathway being involved; (**B**) Western blot analysis for the expression of p-IĸB, p-p65, CyclinD1, Bax, and Bcl-2 in *C20orf27* overexpressing and knockdown cells; (**C**) Flow cytometric analysis for the G1 phase of cells with *C20orf27* overexpression and silencing. * *p* < 0.05, ** *p* < 0.01.

To better understand the molecular mechanisms by which *C20orf27* regulates CRC, we used DIA (Data-independent Acquistion)-based proteomics to screen for differentially expressed proteins between DLD-1/HCT15-NC and DLD-1/HCT15-*C20orf27* cells. IPA (Ingenuity Systems, Redwood City, CA, USA) was then applied to analyze the differentially expressed proteins, showing the upregulation of the NFĸB pathway in response to *C20orf27* overexpression (Figure 2A). To verify whether *C20orf27* regulates the growth and proliferation of CRC via NFĸB, we examined the expression of proteins related to the NFĸB pathway. In HCT15 and DLD-1 cells, after overexpression of *C20orf27*, phosphorylated IĸB, phosphorylated p65, CyclinD1, and Bcl-2 increased, while Bax and cleaved-caspase3 decreased (Figure 2B and Figure S2B). Correspondingly, in SW480 and HT29 cells, silencing of *C20orf27* was positively correlated with the expression of p-IĸB, p-p65, CyclinD1, and Bcl-2, but negatively correlated with Bax and cleaved-caspase3 levels (Figure 2B and Figure S2C). These data indicate that *C20orf27* regulates the cell cycle and apoptosis of CRC cells by activating NFĸB signaling.

**Figure 3:**
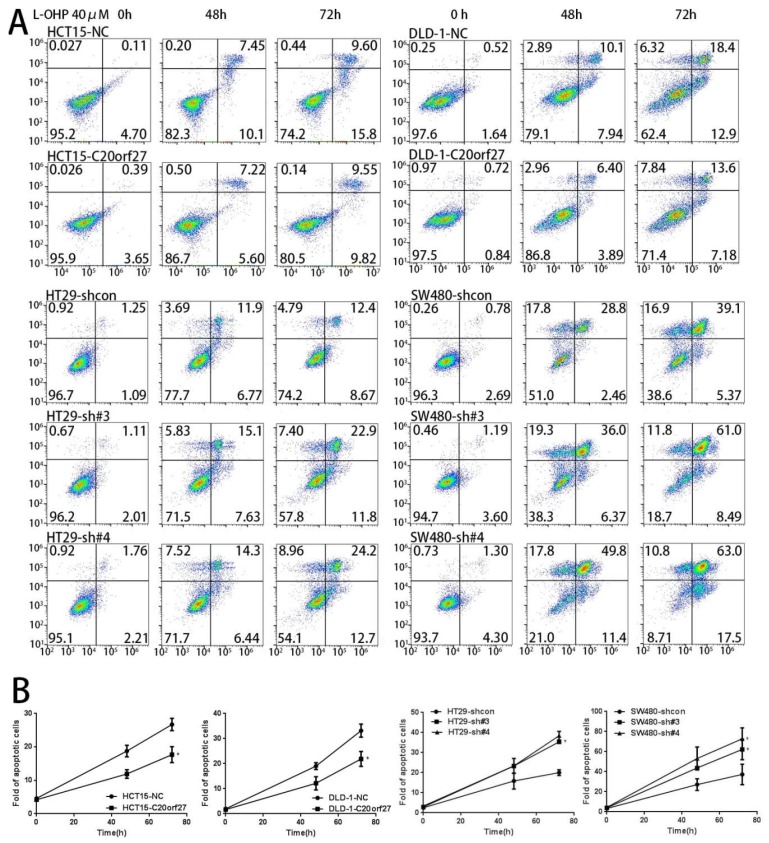
*C20orf27* regulates apoptosis by activating the NFĸB pathway. (**A**) Cells were pretreated with L-OHP (40 μM) for 48 and 72 h. Flow cytometry analyzed apoptosis in *C20orf27* overexpression and silencing cells; (**B**) Quantitative analysis of the proportion of apoptotic cells. * *p* < 0.05.

Then, we used flow cytometry to detect changes in the G1 phase between *C20orf27* overexpressing cells and control cells. The results showed that *C20orf27* reduced the G1 phase of the cells, thereby pushing the cell cycle into a proliferative state. The same conclusion was obtained in SW480 and HT29 silencing cells and controls (Figure 2C,D). To further clarify the effect of *C20orf27* on apoptosis in CRC cells, we used flow cytometry to detect apoptosis at 48 and 72 h of oxaliplatin (L-OHP) treatment (Figure 3A,B). In HCT15 and DLD-1 cells, *C20orf27* overexpression was compared to its control, and the number of apoptotic cells decreased at 48 and 72 h. In SW480 and HT29 cells, *C20orf27* silencing significantly increased apoptosis after 48 and 72 h of oxaliplatin treatment. These data demonstrate that *C20orf27* regulates the cell cycle and apoptosis by activating NFĸB pathways in CRC cells, thereby affecting cell growth and proliferation.

### 2.3. NFĸB Inhibitor Reverses the Growth-Promoting Effect of C20orf27 in CRC Cells

**Figure 4:**
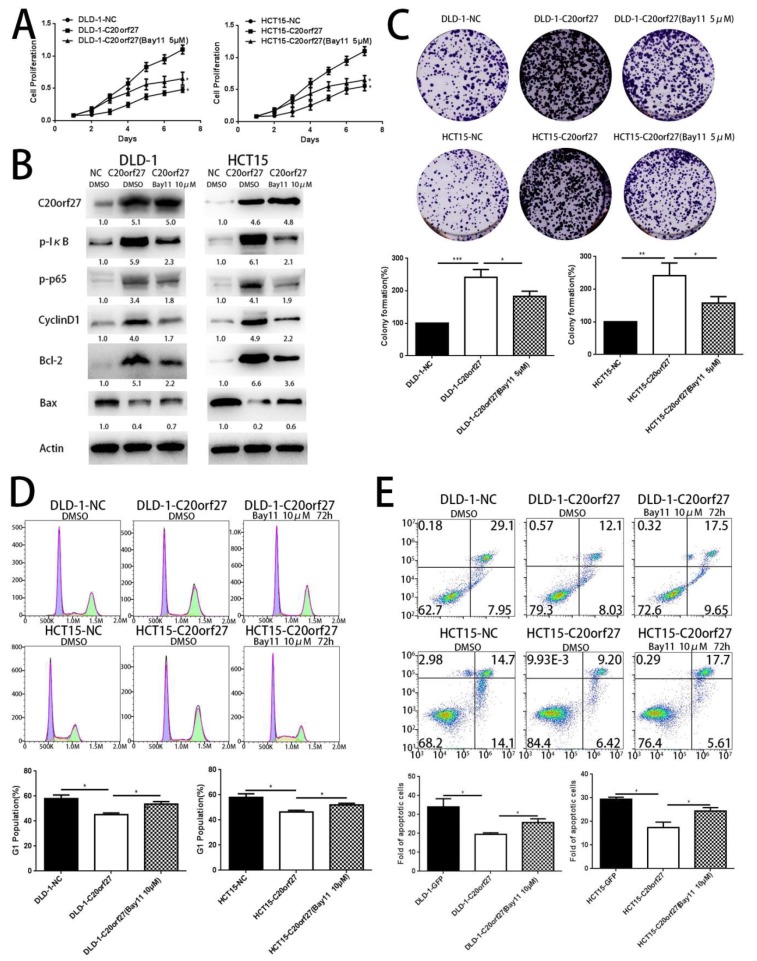
NFĸB inhibitor reverses the growth-promoting effect of *C20orf27* in CRC cells. *C20orf27*-overexpression cells were pretreated with Bay11-7082, and DMSO was used as a control. (**A**) WST-1 analysis for cell proliferation activity; (**B**) Western blot analysis for the expression of p-IĸB, p-p65, CyclinD1, Bax, and Bcl-2; (**C**) Clonal formation assay of cell clonal proliferation ability and quantification; (**D**) Flow cytometry analysis of the cell cycle and quantification. (**E**) Cells were pretreated with L-OHP (40 µM) and Bay11-7082 (10 µM) for 72 h. Flow cytometry analyzed apoptosis in *C20orf27* overexpressing cells. * *p* < 0.05, ** *p* < 0.01, *** *p* < 0.001.

We then used the NFĸB pathway inhibitor Bay11-7082 to further clarify the association of NFĸB with *C20orf27*. WST-1 results showed that, from the third day, Bay11-7082 inhibited the increased growth viability of *C20orf27* overexpressing cells (Figure 4A). The decreased expression of p-IĸB, p-p65, CyclinD1, and Bcl-2 proteins confirmed that Bay11-7082 successfully inhibited the NFĸB pathway in *C20orf27* overexpressing cells (Figure 4B). As shown in Figure 4C, Bay11-7082 also significantly inhibited the clonal proliferation of *C20orf27* overexpressing cells. Subsequently, we also found that treatment with Bay11-7082 increased the G1 phase of HCT15-*C20orf27* and DLD-1-*C20orf27* cells (Figure 4D). The inhibition of apoptosis by *C20orf27* was reversed after 72 h of oxaliplatin treatment in *C20orf27* overexpressing cells of HCT15 and DLD-1 (Figure 4E). Collectively, these data indicate that *C20orf27* regulates the cell cycle and apoptosis via the NFĸB pathway to promote CRC cell growth and proliferation.

### 2.4. C20orf27 Interacts with PP1c to Activate NFĸB through the TGFβR-TAK1 Pathway

**Figure 5:**
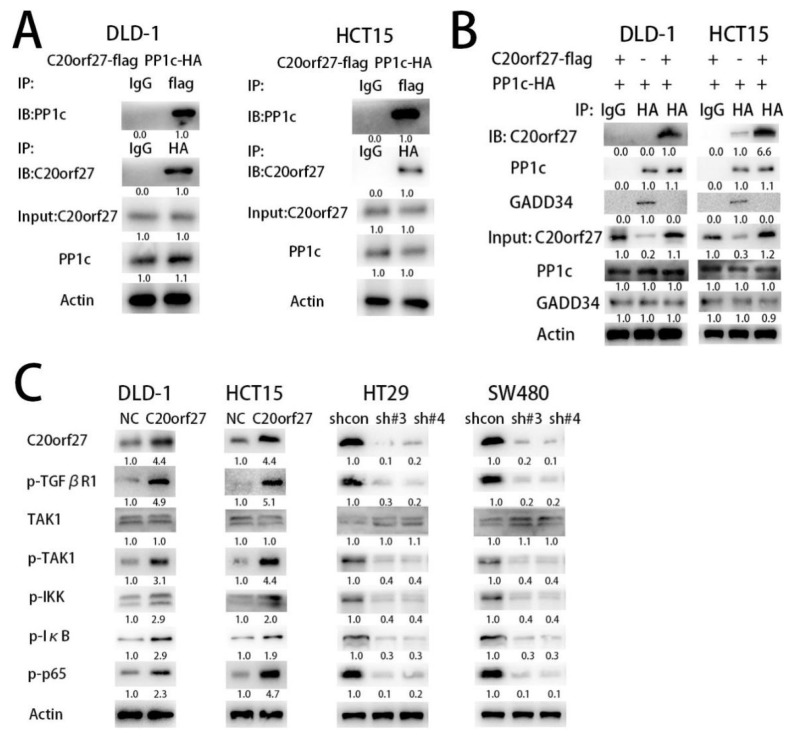
*C20orf27* activates thee TGFβR-TAK1-NFĸB pathway through interaction with PP1c. (**A**) PP1c binding with *C20orf27* was validated by CoIP assay; (**B**) HA-tagged PP1c plasmid was transfected into the cells with or without *C20orf27*-Flag overexpression. CoIP demonstrated that the interaction between PP1c and GADD34 was reduced with *C20orf27* overexpression; (**C**) Western blot analysis for the expression of p-TGFβR1, p-IĸB, p-TAK1, p-IKK, and p-p65 in *C20orf27* overexpressing and silencing cells.

To further investigate the mechanism by which *C20orf27* activates the NFĸB pathway, we used CoIP to search for proteins that interact with *C20orf27*. As shown in Supplementary Materials Figure S2D, we obtained a band that interacts significantly with *C20orf27* at the 37 kDa position. According to the mass spectrometry results, the band is the catalytic subunit of serine threonine (PP1c) [8]. To further confirm whether *C20orf27* interacts with PP1c, the *C20orf27* and PP1c plasmids with the marker were transfected into HCT15 and DLD-1 cells. CoIP experiments demonstrated that *C20orf27* and PP1c can be co-precipitated, indicating an interaction between the two proteins (Figure 5A).

To further support this conclusion, we transiently transfected the HA-tagged PP1c plasmid into the cells with or without *C20orf27*-Flag overexpression and then performed CoIP assay. As shown in Figure 5B, the interaction between PP1c and the regulatory subunit GADD34 was reduced with *C20orf27* overexpression. Therefore, we conclude that *C20orf27* inhibits the recruitment of PP1c to its regulatory subunit by binding to PP1c, thereby inhibiting the phosphatase action of PP1.

In mammals, the receptor serine threonine kinase family is a receptor for TGF (Transforming growth factor) family ligands [9]. Phosphorylation of TGFβR induces the sequential phosphorylation of TAK1 (Transforming growth factor-β activated kinase-1), IKK (inhibitor of nuclear factor kappa-B kinase), IĸBa (inhibitor kappa-Bα), and RelA (NF-kB subunit), and TAK1 enhances NFĸB activation [10]. In this regard, we verified the phosphorylation status of proteins related to the TGFβR-TAK1-NFĸB pathway. As shown in Figure 5C, with *C20orf27* overexpression in HCT15 and DLD-1 cells, the expression of p-TGFβR1, p-TAK1, p-IKK, p-IĸB, and p-p65 was increased. Reversely, in HT29 and SW480 cells with *C20orf27* silencing, p-TGFβR1, p-TAK1, p-IKK, p-IĸB, and p-p65 expressions were reduced. These data indicate that *C20orf27* inhibits the formation of PP1 holoenzyme by binding to PP1c, thereby suppressing the inhibitory effect of PP1 on the TGFβR-TAK1-NFĸB pathway. In other words, *C20orf27* activates the TGFβR-TAK1-NFĸB pathway, promoting tumor cell growth and proliferation in CRC.

### 2.5. C20orf27 Promotes Tumor Growth In Vivo

**Figure 6:**
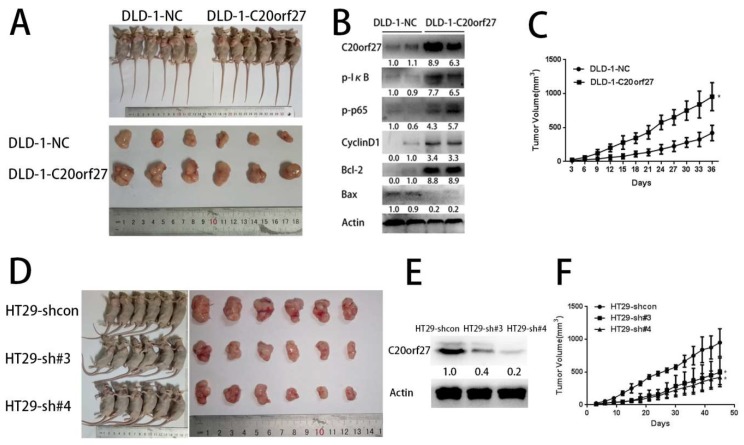
*C20orf27* promotes tumor growth and proliferation in vivo. (**A**) Formation of tumors in mice injected with *C20orf27* overexpression and control cells; (**B**) Western blot analysis in tumor tissues; (**C**) Tumor volume of nude mice with *C20orf27* overexpression and control cells during the experiment; (**D**) Formation of tumors in mice injected with *C20orf27* silencing and control cells; (**E**) Western blot analysis in tumor tissues; (**F**) Tumor volume of nude mice with *C20orf27* silencing and control cells during the experiment. * *p* < 0.05.

To further confirm the role of *C20orf27* in driving CRC growth, in vivo experiments with subcutaneous tumor formation in nude mice were also performed. Compared with the control group, CRC cells with stably expressing *C20orf27* had superior tumor growth ability after subcutaneous injection of cells, which was supported by changes in the tumor volume (Figure 6A–C). As shown in Figure 6B, we detected the NFĸB pathway-related proteins in nude mice tumor cells. The results showed that *C20orf27* was positively correlated with phosphorylated IĸB, phosphorylated p65, CyclinD1, and Bcl-2, which was consistent with the results from the in vitro experiments. Reversely, CRC cells with stable silencing of *C20orf27* had a weaker ability in tumor growth as compared to control cells (Figure 6D–F). These observations indicate that *C20orf27* plays a role in the regulation of CRC tumor formation and growth.

## 3. Discussion

**Figure 7:**
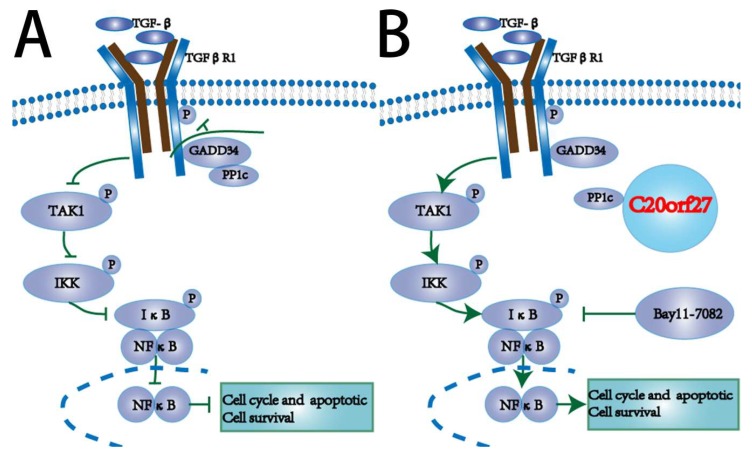
Schematic mechanism. (**A**) Binding of PP1c and GADD34 inhibits the TGFβR-TAK1-NFĸB pathway; (**B**) *C20orf27* interacts with PP1c, releasing GADD34 to activate the TGFβR-TAK1-NFĸB pathway, promoting tumor growth in CRC.

CRC is still a malignant tumor that seriously threatens human life. Therefore, it is necessary to continue our efforts in finding tumor markers and key genes for the diagnosis and treatment of CRC. In this study, we identified the functionally unknown gene *C20orf27* on chromosome 20 of the human genome, and studied its role and regulation mechanism in the development of CRC. We found that overexpression of *C20orf27* promoted CRC cell growth and proliferation, and *C20orf27* silencing reduced the clonal formation and growth ability of CRC in vitro. Further analysis indicated that *C20orf27* regulates the cell cycle and apoptosis by activating the NFĸB pathway in CRC, thereby promoting cell proliferation. We then revealed that *C20orf27* inhibits the phosphatase action of PP1 holoenzyme in the TGFβR-TAK1-NFĸB pathway by interacting with PP1c, thus activating NFĸB (Figure 7). Subcutaneous injection of CRC cells into nude mice verified the functionality of *C20orf27* in promoting tumor formation and growth.

As the key player in the TGFβR-TAK1-NFĸB pathway involved, the TGF family regulates cell survival by regulating apoptosis and proliferation, and therefore plays an important role in the health or disease processes of different tissues [11]. Obstacles in the signaling of the TGF family can cause a variety of human diseases, including malignant tumors and autoimmune diseases [11,12]. The transmission of TGF-β signaling is regulated by a heterotetrameric complex containing two transmembrane receptor serine/threonine kinases whose activity is controlled by serine/threonine phosphorylation [9,11]. The TGF signaling pathway is initiated by a ligand that binds to a type II receptor (TGFβRII) and then forms a receptor complex with a type I signaling receptor (TGFβRI) to start an intracellular response [9].

Mammalian kinases/phosphatases include two members, one is a protein tyrosine kinase/phosphatase and the other is a serine threonine kinase/phosphatase (PP) [9]. The phosphorylation and dephosphorylation states of cellular proteins are regulated by the opposite effects of protein kinases and phosphatases. The catalytic subunit PP1c of type 1 phosphatase (PP1) is recruited to the TGFβR1-Smad7-GADD34 complex via its regulatory subunit GADD34, thereby dephosphorylating TGFβRI [9]. The PP1 holoenzyme produces dephosphorylation of TGFβR, which forms a negative feedback in the TGF signaling pathway [9]. Dephosphorylation of TGFβRI is an effective mechanism for controlling negative feedback in the process of TGF pathway conduction. The TGFβ signaling pathway inhibits the phosphorylation of TAK1 by recruiting PP1c through the TGFβR1-Smad7-GADD34 complex, thereby inhibiting the phosphorylation of IKK and IĸB and further inhibiting the nuclear translocation of NFĸB [9,10].

NFĸB transcription factors are key regulators of many cellular processes, including cellular immunity, cell proliferation and apoptosis, cellular inflammation, and acute phase responses in cells [13]. In the process of the occurrence and development of malignant tumors, the signal pathway capable of controlling cell growth and proliferation is crucial. In most normal cells, these NFĸB dimers bind directly to the IĸB inhibitor and remain in the cytoplasm as an inactive complex [14,15]. Many signaling pathways may cause the degradation of the IĸB protein and activate the NFĸB complex, which is transferred to the nucleus [14,15]. NFĸB regulates the cell cycle through cyclinD1, and regulates apoptosis through Bcl-2 and caspase3, thereby affecting the survival and proliferation of tumor cells [16]. Our study revealed the regulation of *C20orf27* on the TGFβR-TAK1-NFĸB pathway in CRC cells. The binding of *C20orf27* to PP1c was confirmed, which impaired the dephosphorylation inhibition of PP1 on this pathway, thereby activating NFĸB and exerting its regulatory effects on the cell cycle and apoptosis. The current data clearly demonstrated that *C20orf27* promotes the growth and proliferation of CRC cells by regulating the TGFβR-TAK1-NFĸB pathway.

In addition, according to the results obtained in the oxaliplatin treatment (Figure 3), *C20orf27* may be related to drug resistance and thus may be considered for further exploration as a marker of prognosis. This will be a focus of our next investigation.

## 4. Materials and Methods

### 4.1. Cell Lines and Culture

The cells including NCM460, SW480, SW620, RKO, HCT15, HCT116, DLD-1, and HT29 were all grown in 1640 (RPMI-1640) medium containing 10% fetal bovine serum (Gibco, Grand Island, NY, USA), while Caco2 was grown in DMEM (dulbecco’s modified eagle medium) (Gibco, Grand Island, NY, USA) medium containing 10% fetal bovine serum. All these cells were cultured in an incubator containing 5% carbon dioxide at 37 °C.

### 4.2. Transfection

Stable transfection: The sequences were transfected into 293T cells, packaged using a lentiviral packaging mix, and used to infect CRC cells to establish cells that constitutively inhibit or express *C20orf27*. Stable clones were selected with puromycin (1 μg/mL).

Transient transfection: Cells were grown in RPMI-1640 medium to 50% and then transfected using Lipofectamine 3000 (Life Technologies, corporation Gaithersburg, MD, USA) according to the manufacturer’s instructions.

### 4.3. Western Blot Analysis

The cells were lysed for 30 min and then centrifuged at 13,000× *g* for 30 min at 4 °C. The quantified protein was mixed in proportion with the protein loading buffer and boiled at 95 °C for 10 min. The sample was electrophoresed and then transferred to a polyvinylidene difluoride (PVDF) membrane. The skim milk powder was diluted with Tween-20 Tris buffered saline (TBST) for 1 h at room temperature, then incubated with the primary antibody overnight, washed with TBST, and then incubated with the corresponding secondary antibody for 1 h at room temperature. The reaction was visualized using electrochemiluminescence (ECL, Bio-Rad, Hercules, CA, USA) and detected by exposure to autoradiographic film. The densitometric reading/intensity ratio for each strip was included in all western blots (Figures S3 and S4).

The primary antibodies used included CyclinD1 from BD Pharmingen(SanDiego, CA, USA); p-IĸB, p-NFĸB1, Bcl-2, Bax, p-TAK1, p-IKK, caspase3, and cleaved caspase3 from Cell Signaling Technology(Danvers, MA, USA); actin from Transgen Biotech(Beijing, China); *C20orf27* and p-TGFβR1 from Abcam(Cambridge, MA, USA); Bay11-7082 from TargetMol(Boston, MA, USA); and PP1c, GADD34, and TAK1 from Santa Crus (CA, USA).

### 4.4. Real-Time QRT-PCR

Total RNA was extracted by the Trizol method, 200 μL of chloroform was added, vortexed to pink, dissolved on ice for 30 min, centrifuged at 12,000× *g* for 15 min, 500 μL of supernatant was carefully aspirated, isopropanol was added, placed on ice for 30 min, centrifuged at 12,000× *g* for 15 min, and removed. Then, pre-cooled 75% ethanol was added to the precipitate, mixed, and centrifuged at 12,000× *g* for 7 min. The RNA precipitate was diluted with water, mixed, and the concentration was measured.

The extracted RNA was reverse transcribed into cDNA. Primers were purchased from Ruibiotech (Guangzhou, China). The 20-μL system consisted of 1 μL of each of the upstream and downstream primers, 1 μL of the cDNA template, 7 μL of ultrapure water, and 10 μL of mix. After shaking and mixing, 20 μL was dispensed into the qPCR tube. Real-time PCR was performed on an Applied Biosystems StepOne Real-Time PCR system (Thermo Fisher Scientific, Waltham, MA, USA). The reaction conditions were set to 95 °C pre-denaturation for 30 s, 95 °C denaturation for 0.05 s, 60 °C renaturation for 30 s, 45 cycles.

### 4.5. Colony-Formation Assay

In total, 2000 cells were cultured in a 6-well plate for 2 weeks. After 2 weeks, cells were washed once using PBS (phosphate-buffered saline), fixed with a certain amount of methanol for 10 min, added with crystal violet for 5 min, rinsed, dried, and scanned.

### 4.6. Flow Cytometric Assay

After collecting the cells, the cells were fixed by adding pre-cooled ethanol at 4 °C for 2 h, resuspended in 300 μL of PBS containing 10% FBS, and fixed by adding 700 μL of pre-cooled absolute ethanol overnight at −20 °C. The next day, centrifugation was done at 300× *g* for 5 min, and the ethanol was removed. The cells were resuspended in 300 μL of PI (propidium iodide) staining solution (Beyotime, Shanghai, China), and after 15 min of reaction, they were filtered. The cell cycle was detected by flow cytometry (BD AccuriC6, Franklin Lakes, NJ, USA).

After the cells and cell supernatant were collected, the cells were washed twice with PBS. After collecting 10^5^–5 × 10^5^ cells, 100 μL binding buffer, 1 μL Annexin V-FITC (Biyotime), and 1 μL propidium iodide were added to the cells. The cells were allowed to stand for 5 to 15 min at room temperature and apoptosis was detected by flow cytometry.

### 4.7. Tumorigenicity in Nude Mice

Female nude mice of 4-6 weeks were purchased, with 8 in each group. The cells were collected, washed 2–3 times with PBS, and then resuspended in PBS. Then, 5 × 10^6^ CRC cells were injected subcutaneously into the animals. The body weight and tumor size of nude mice were measured daily. The study was approved by the institutional review board of Jinan University (Ethical number: 20180705-19).

The biomedical research involving humans in this institute was approved by the Ethics Committee of the First Affiliated Hospital of Jinan University (Ethics Number: 20190228).

### 4.8. Co-Immunoprecipitation (CoIP) Assay

Cells were collected and lysed with IP (immunoprecipitation) lysates on ice for 40 min, and then centrifuged at 13,000 rpm for 4 min at 40 °C. The supernatant was collected, and the protein concentration was measured by the BCA (bicinchoninic acid) method. Immunoglobulin G (IgG, 2 μg) was added to 1 mg of protein, mixed with 20 μL of protein A/G Sepharose beads for 1 h in a homomixer, and then centrifuged at 2500 rpm for 5 min. The supernatant was collected, 1 mg of protein was added to 2 μg of the target protein antibody, and the mixture was mixed overnight. Protein A/G Sepharose (20 μL) was added to the protein and mixed for 4 h in a homomixer. Beads were washed with PBS 3 times, the supernatants were collected, and Western blot analysis was conducted.

### 4.9. Cell Viability Assay

The cells were seeded in a 96-well plate at 100 μL per well (1000 cells) and cultured overnight. Then, 10 μL of WST-1 (Biyotime) was added to each well, and protected from light. After incubation for 3 h in a cell culture incubator, the absorbance (450 nm) was measured with a microplate reader for 7 days, and the absorbance was measured at the same time every day.

### 4.10. MS and Bioinformatics Analyses

Whole-cell lysates were homogenized in RIPA (Radio-Immunoprecipitation Assay) lysis buffer. After adding urea and DTT (DL-Dithiothreitol) to the protein samples, the protein samples were bathed at 37 °C for 1 h. Protein samples were added to IAA (indole-3-acetic acid) and allowed to stand at room temperature for 30 min. The sample was added to an ultrafiltration tube and centrifuged at 12,000× *g* for 15 min. After further digestion with trypsin, the samples were lyophilized and resuspended in anhydrous acetonitrile solution. The samples were desalted, and the peptide samples were analyzed using an Orbitrap Fusion Lumos mass spectrometer (Thermo Fisher Scientifc, Waltham, MA, USA). The differently expressed proteins were analyzed by Ingenuity Pathway Analysis (IPA, Ingenuity Systems, Redwood City, CA, USA).

### 4.11. Statistical Analysis

The data were statistically analyzed using Student’s t-test with GraphPad Prism 6.0 (La Jolla, CA, USA), and *p* < 0.05 was considered to indicate significant differences. All experiments were repeated three times, and representative data are shown as * *p* < 0.05, ** *p* < 0.01, *** *p* < 0.001.

## 5. Conclusions

This report for the first time illustrated the functional role of a previously functionally unknown gene *C20orf27* in CRC and its molecular mechanisms in vitro and in vivo. *C20orf27* plays an important role in driving the growth and proliferation of CRC via the TGFβR-TAK1-NFĸB pathway. *C20orf27* inhibits the formation of PP1 holoenzyme by interacting with PP1c, thus reducing the inhibitory effect of PP1 on the TGFβR-TAK1-NFĸB pathway. Considering the powerful functionality of *C20orf27* in promoting the malignant progression of CRC, *C20orf27* can be further explored as a tumor marker and a novel therapeutic target for CRC.

## Supplementary Materials

The following are available online at https://www.mdpi.com/2072-6694/12/2/336/s1, Figure S1: Screening of unknown functional genes on chromosome 20, Figure S2: *C20orf27* interacts with PP1c to activate NFĸB through TGFβR-TAK1 pathway, Figure S3. The whole blot showing all the bands with all molecular weight markers on the Western blotting, Figure S4. The whole blot showing all the bands with all molecular weight markers on the Western blotting.

## References

[1] Dekker E., Tanis P.J., Vleugels J.L.A., Kasi P.M., Wallace M.B. (2019). Colorectal cancer. Lancet.

[2] Bray F., Ferlay J., Soerjomataram I., Siegel R.L., Torre L.A., Jemal A. (2018). Global cancer statistics 2018: GLOBOCAN estimates of incidence and mortality worldwide for 36 cancers in 185 countries. CA Cancer J. Clin..

[3] Arnold M., Sierra M.S., Laversanne M., Soerjomataram I., Jemal A., Bray F. (2017). Global patterns and trends in colorectal cancer incidence and mortality. Gut.

[4] Moraes F., Goes A. (2016). A decade of human genome project conclusion: Scientific diffusion about our genome knowledge. Biochem. Mol. Biol. Educ..

[5] Skrzypczak M., Goryca K., Rubel T., Paziewska A., Mikula M., Jarosz D., Pachlewski J., Oledzki J., Ostrowsk J. (2010). Modeling oncogenic signaling in colon tumors by multidirectional analyses of microarray data directed for maximization of analytical reliability. PLoS ONE.

[6] Hong Y., Downey T., Eu K.W., Koh P.K., Cheah P.Y. (2010). A ‘metastasis-prone’ signature for early-stage mismatch-repair proficient sporadic colorectal cancer patients and its implications for possible therapeutics. Clin. Exp. Metastasis.

[7] Gaedcke J., Grade M., Jung K., Camps J., Jo P., Emons G., Gehoff A., Sax U., Schirmer M., Becker H. (2010). Mutated KRAS results in overexpression of DUSP4, a MAP-kinase phosphatase, and SMYD3, a histone methyltransferase, in rectal carcinomas. Genes Chromosomes Cancer.

[8] Huttlin E.L., Ting L., Bruckner R.J., Gebreab F., Gygi M.P., Szpyt J., Tam S., Zarraga G., Colby G., Baltier K. (2015). The BioPlex Network: A Systematic Exploration of the Human Interactome. Cell.

[9] Shi W.B., Sun C.X., He B., Xiong W.C., Shi X.M., Yao D.C., Cao X. (2004). GADD34–PP1c recruited by Smad7 dephosphorylates TGF type I receptor. J. Cell Biol..

[10] Freudlsperger C., Bian Y., Wise S.C., Burnett J., Coupar J., Yang X., Chen Z., Waes C.V. (2013). TGF-b and NF-kB signal pathway cross-talk is mediated through TAK1 and SMAD7 in a subset of head and neck cancers. Oncogene.

[11] Attisano L., Wrana J.L. (2002). Signal transduction by the TGF-superfamily. Science.

[12] Derynck R., Akhurst R.J., Balmain A. (2001). TGF-signaling in tumor suppression and cancer progression. Nat. Genet..

[13] Rayet B., Gelinas C. (1999). Aberrant rel/nfkb genes and activity in human cancer. Oncogene.

[14] Pahl H.L. (1999). Activators and target genes of Rel/NF-kappaB transcription factors. Oncogene.

[15] Karin M. (1999). How NF-kappaB is activated: The role of the IkappaB kinase (IKK) complex. Oncogene.

[16] Liu T., Zhang L.Y., Joo D.Y., Sun S.C. (2017). NFκB signaling in inflammation. Signal Transduct. Target. Ther..

