# Supplementary material for: C20orf27 Promotes Cell Growth and Proliferation of Colorectal Cancer via the TGFβR-TAK1-NFĸB Pathway
Source: Cancers (Basel). 2020 Feb 2;12(2):336. doi: 10.3390/cancers12020336 (PMC7072304; doi:10.3390/cancers12020336)
Supplement: Supplementary file 1 [file cancers-12-00336-s001.pdf]

Supplementary Materials: C20orf27 Promotes Cell Growth and Proliferation of Colorectal Cancer via TGFβR-TAK1-NFκB Pathway

Jing Gao, Yang Wang, Weixia Zhang, Jing Zhang, Shaohua Lu, Kun Meng, Xingfeng Yin, Zhenghua Sun and Qing-Yu He

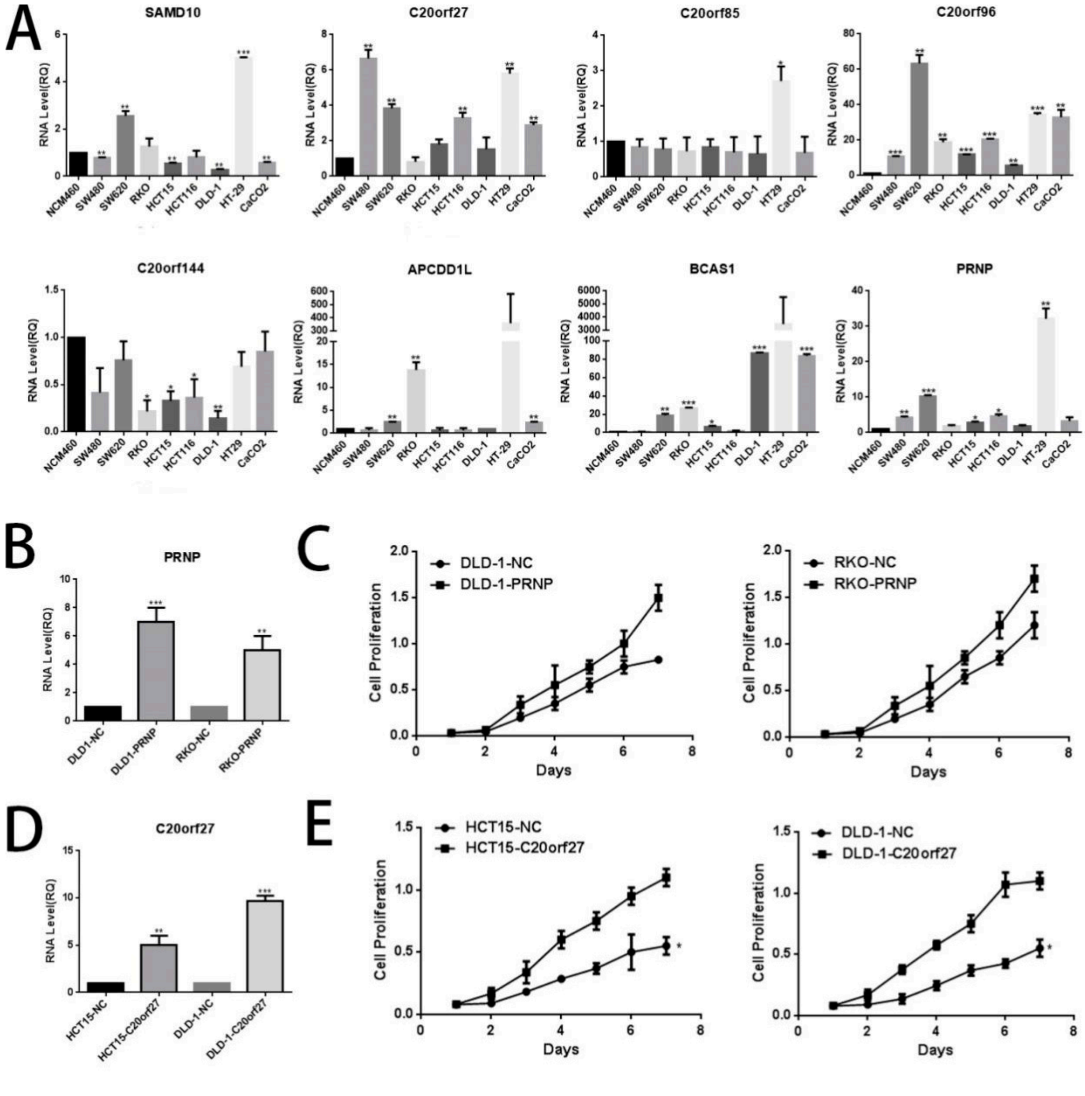

Figure S1. Screening of unknown functional genes on chromosome 20. (**A**) Eight genes were confirmed at the transcriptional level of eight CRC cells and one normal intestinal epithelial cell (NCM460), respectively. (**B**) PRNP was confirmed at the transcriptional level of PRNP overexpressing and control cells. (**C**) Proliferation viability in PRNP overexpression and control cells using WST-1. (**D**) C20orf27 was confirmed at the transcriptional level of C20orf27 overexpressing and control cells. (**E**) Proliferation viability in C20orf27 overexpression and control cells using WST-1.

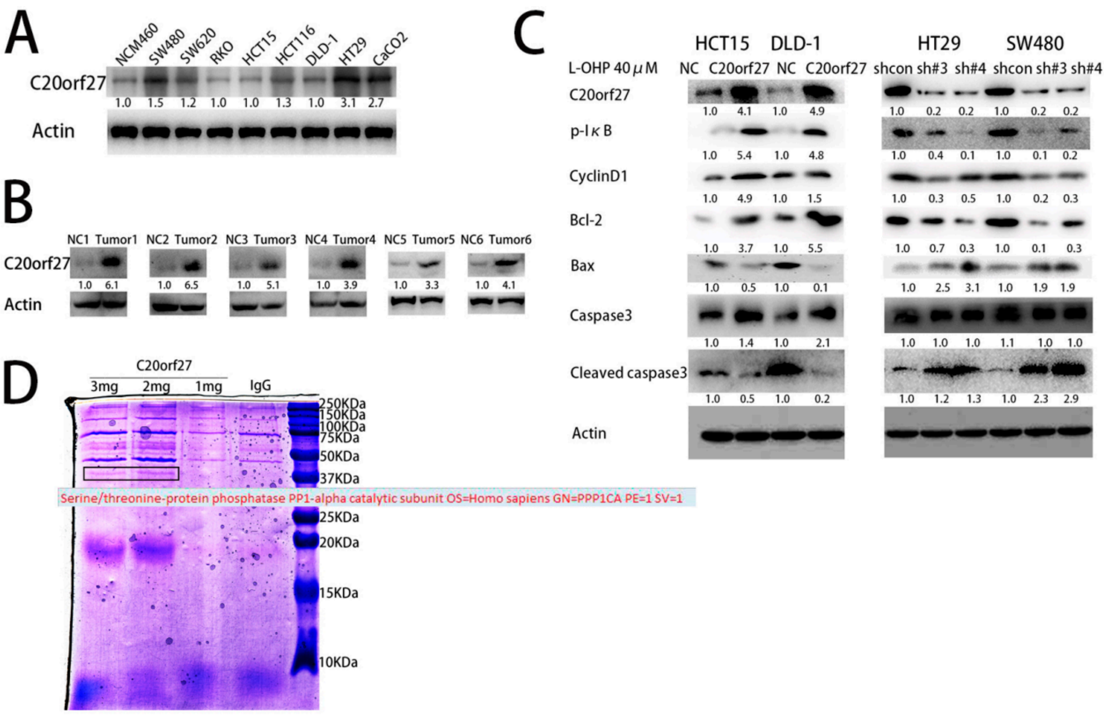

Figure S2. C20orf27 interacts with PP1c to activate NFκB through TGFβR-TAK1 pathway. (**A**) C20orf27 was confirmed at the protein level of eight CRC cells and one normal intestinal epithelial cell (NCM460). (**B**) C20orf27 expression was detected using Western blot analysis in 6 pairs of colorectal cancer tissues and adjacent tissues from patients. (**C**) Cells were pretreated with L-OHP (40 μM) for 72 h. Western blot analysis for the expression of p-IκB, caspase-3, cleaved-caspase-3, p-p65, CyclinD1, Bax, and Bcl-2 in C20orf27 overexpressing and knockdown cells. (**D**) CoIP searches for proteins that interact with C20orf27 and then mass spectrometry was used to identify unknown proteins. The band that significantly interacts with C20orf27 at the 37 kDa position was identified to be the catalytic subunit of serine threonyl phosphatase.

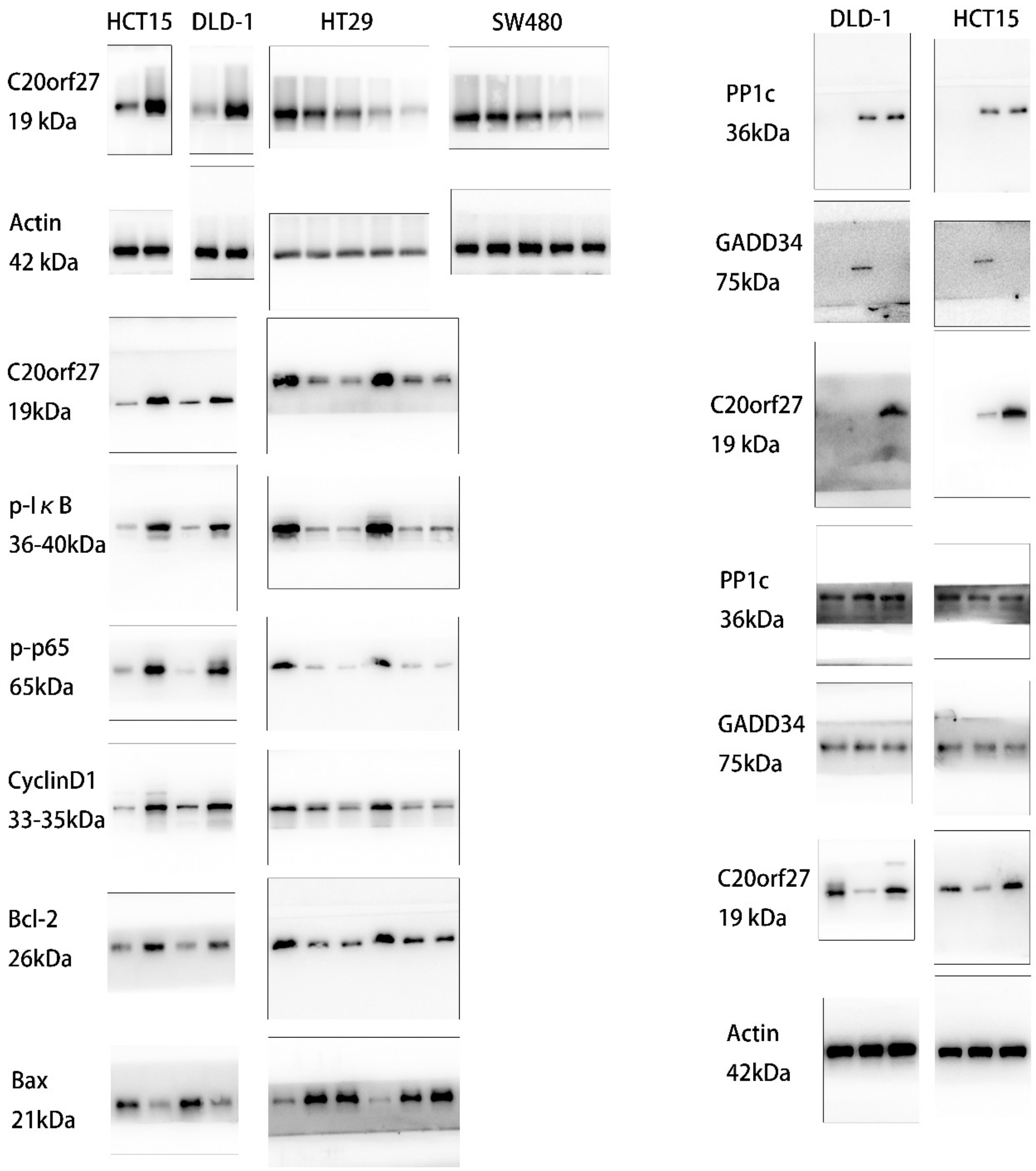

Figure S3. The whole blot showing all the bands with all molecular weight markers on the Western blotting.

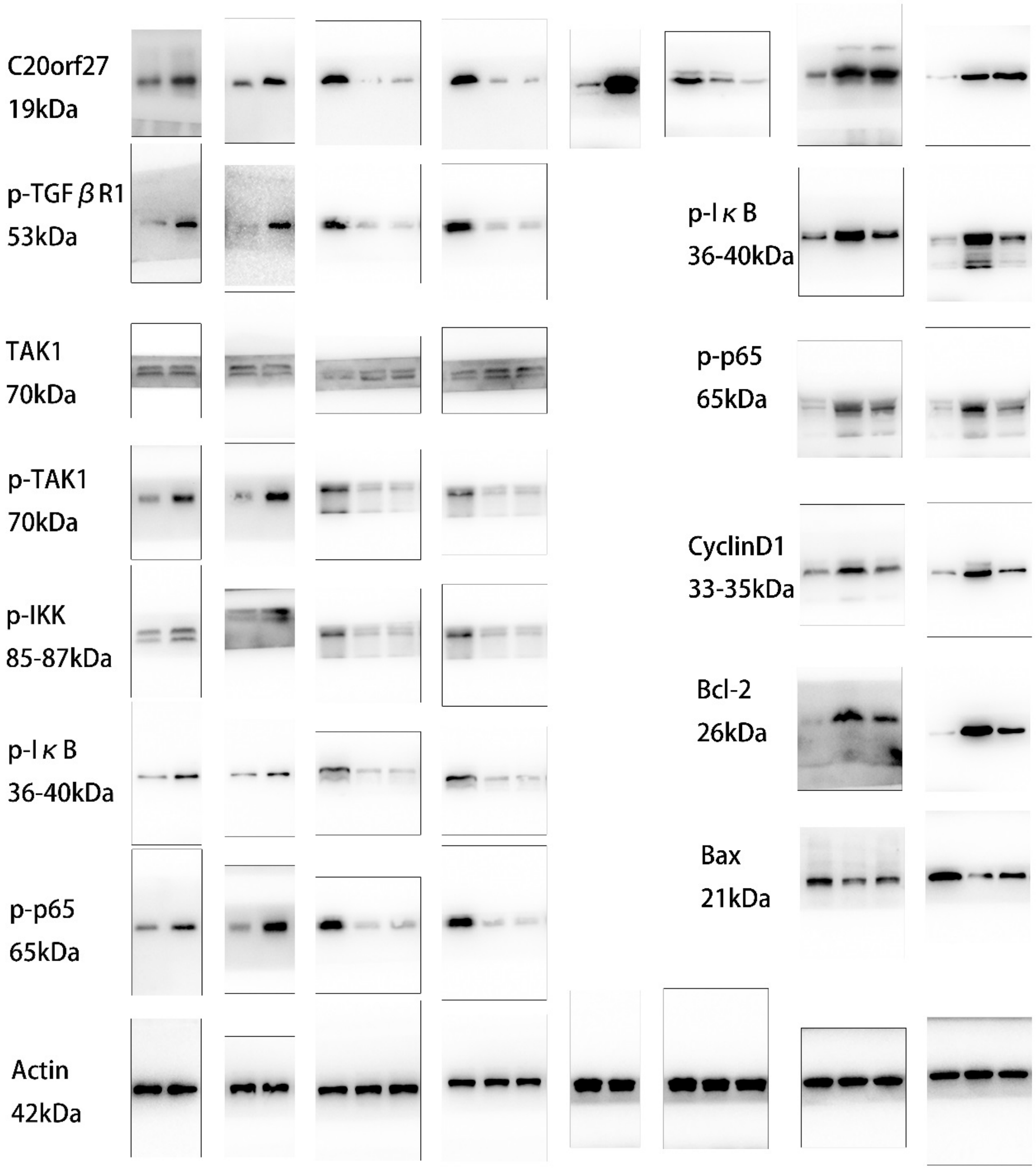

Figure S4. The whole blot showing all the bands with all molecular weight markers on the Western blotting.

© 2020 by the authors. Licensee MDPI, Basel, Switzerland. This article is an open access article distributed under the terms and conditions of the Creative Commons Attribution (CC BY) license (http://creativecommons.org/licenses/by/4.0/).
